# Collision Enhanced Raman Scattering (CERS): An Ultra-High Efficient Raman Enhancement Technique for Hollow Core Photonic Crystal Fiber Based Raman Spectroscopy Gas Analyzer

**DOI:** 10.3390/bios13110979

**Published:** 2023-11-09

**Authors:** Maryam Shirmohammad, Michael A. Short, Haishan Zeng

**Affiliations:** 1 Department of Physics and Astronomy, University of British Columbia, 6224 Agricultural Road, Vancouver, BC V6T 1Z1, Canada; smaryam@umich.edu; 2 Imaging Unit, Integrative Oncology Department, BC Cancer Research Institute, Vancouver, BC V5Z 1L3, Canada; mshort@bccrc.ca; 3 Department of Dermatology and Skin Science, University of British Columbia, Vancouver, BC V5Z 4E8, Canada

**Keywords:** collision-enhanced Raman spectroscopy, hollow-core photonic-crystal fiber, gas analysis, breath analysis, volatile organic compound, Raman scattering

## Abstract

Raman enhancement techniques are essential for gas analysis to increase the detection sensitivity of a Raman spectroscopy system. We have developed an efficient Raman enhancement technique called the collision-enhanced Raman scattering (CERS), where the active Raman gas as the analyte is mixed with a buffer gas inside the hollow-core photonic-crystal fiber (HCPCF) of a fiber-enhanced Raman spectroscopy (FERS) system. This results in an enhanced Raman signal from the analyte gas. In this study, we first showed that the intensity of the 587 cm^−1^ stimulated Raman scattering (SRS) peak of H_2_ confined in an HCPCF is enhanced by as much as five orders of magnitude by mixing with a buffer gas such as helium or N_2_. Secondly, we showed that the magnitudes of Raman enhancement depend on the type of buffer gas, with helium being more efficient compared to N_2_. This makes helium a favorable buffer gas for CERS. Thirdly, we applied CERS for Raman measurements of propene, a metabolically interesting volatile organic compound (VOC) with an association to lung cancer. CERS resulted in a substantial enhancement of propene Raman peaks. In conclusion, the CERS we developed is a simple and efficient Raman-enhancing mechanism for improving gas analysis. It has great potential for application in breath analysis for lung cancer detection.

## 1. Introduction

Raman spectroscopy is a versatile fingerprinting tool for the analysis of gas samples in demanding fields such as breath gas analysis [1,2], petrochemical industries [3,4], and environmental studies [5,6]. Raman spectroscopy and other optical technologies for breath/gas analysis have advantages of lower system costs, compact sizes, and much faster turnaround times as compared to the other popular approach—mass spectrometers [2]. However, Raman gas analysis is challenging due to low-molecular-number densities in addition to the intrinsically low inelastic Raman scattering cross-sections. Approximately only one in a million interactions results in Raman scattering [7,8]. Therefore, Raman enhancement techniques are crucial for gas analysis. Various Raman enhancement techniques have been developed and applied to improve Raman signal intensity from gases.

In cavity-enhanced Raman spectroscopy, the effective pathlength for Raman interaction is increased through multiple-beam passing by using specialized reflection mirrors in the gas cell [9,10,11,12].

Nonlinear enhancement techniques such as coherent anti-Stokes Raman scattering (CARS) and stimulated Raman scattering (SRS) utilize multiple-pump-Stokes-pulsed lasers to coherently drive the vibrational or rotational modes of gas molecules, with Raman intensities’ orders of magnitude greater than conventional spontaneous Raman scattering [13,14,15].

Fiber-enhanced Raman spectroscopy (FERS) [2,16] is relatively a new enhancement technique for gas analysis, which utilizes specialized hollow-core optical fibers designed with minimal attenuation losses. These fibers include the bandgap transmission hollow-core fibers [17,18] and anti-resonant hollow-core fibers [19,20]. The use of these hollow-core fibers increases the probability of Raman interactions by providing a tight confinement of the sample for light and gas interactions; therefore, increasing the physical length of the fiber increases the light–gas interaction pathlength without the significant attenuation of the Raman signal. FERS systems incorporating bandgap fibers, such as hollow-core photonic-crystal fibers (HCPCFs) [2,21,22] or anti-resonant hollow-core fibers, have been increasingly reported for their sensitive gas analysis applications.

HCPCFs are highly efficient for the enhancement of Raman scattering intensities. HCPCF-based FERS systems are frequently used for gas mixture components analysis in environmental and petrochemical industries [23,24,25] and for breath analysis for disease monitoring and diagnosis [16,26]. Our group is one of the two pioneers [2,16] of FERS system development for breath analysis using continuous wave (CW) laser as the Raman excitation pump with promising performance. Major components of exhaled-breath gas were successfully identified with our FERS system; however, further improvement on the detection sensitivity of the system is required to identify trace amounts of components in breath, such as VOCs.

Raman spectroscopy-based breath analysis is a promising and powerful technique for a non-invasive diagnosis of various diseases with specific biomarkers in the breath. These novel applications of FERS gas analyzers require an ultra-high sensitivity detection of breath biomarkers. Traditional ways of increasing the sensitivity of a fiber-based Raman gas analyzer are to increase the pump power, increase the interaction fiber length, or concentrate the analyte. But, these methods all have their own drawbacks. For example, increasing the excitation power might produce thermal shock and damage the fiber, considering inevitable drifts in the laser–fiber alignment during the course of measurements [27]. The filling and emptying time of the fiber increases substantially with fiber length [28], which affects the throughput of analysis. Breath sampling necessary for purification and concentration can impact the accuracy of VOC analysis [29]. As such, the field will benefit from innovations in Raman enhancement technique that can add on to the enhancements already provided by the FERS systems.

Benabid et al. showed that when H_2_ atoms were confined in an HCPCF and pumped with nanosecond pulses, the SRS of first rotational Stokes line of H_2_ was generated with much smaller energy thresholds than those previously reported [30]. The pump power reduction for SRS from H_2_ in an HCPCF was reported to be two orders of magnitude lower for vibrational transitions [30], and six orders of magnitude lower for rotational transitions [31] compared to the previously reported values. The same group published numerous reports on the generation of new laser wavelengths based on Raman transitions with confining gas particles inside HCPCF core and pumping with high-energy-pulsed lasers [31,32,33].

Incorporating a pulsed pump with an FERS system for the purpose of gas Raman spectra enhancement through SRS has not been explored prior to our work. The initial main motivation of our work was to develop a Raman enhancement technique for gas analysis through an SRS-capable FERS system, which was realized by replacing the CW pump with a high-energy nanosecond-pulsed pump in our already developed FERS system. We demonstrated that this had led to an efficient improvement to the measured Raman signal intensity [34], which is useful for gas analysis and can be applied to the components of breath gas.

Interestingly, we observed an unexpected Raman-enhancing phenomenon while performing characterization tests of our SRS-capable FERS system with a simple two-component simulation breath gas sample. Mixtures of H_2_–N_2_ were prepared to simulate a simple two-component exhaled breath gas with the thought that N_2_ would not affect Raman intensity measurements of H_2_, as a vibrational Raman shift of N_2_ (2331 cm^−1^) [35] was not in the transmission spectral bandwidth of the HCPCF. N_2_ was chosen as it is abundant in both air and breath. To test this idea, a series of Raman measurements were performed with pure H_2_, followed by mixing with N_2_, keeping the partial pressure of H_2_ unchanged. Our observations were surprising and guided us to designing new sets of Raman measurements toward studying the enhancing effect of a Raman inactive buffer gas mixed with the analyte gas.

We hypothesize that mixing the target gas with a second buffer gas in an HCPCF is an ultra-efficient Raman enhancement technique, which is associated with the collisions between the two gas particles; hence, collision-enhanced Raman scattering (CERS) is the term to refer to for this enhancement mechanism. To the best of our knowledge, CERS has not been reported as an enhancement technique for the Raman spectroscopy analysis of gas samples in a hollow-core photonic-crystal fiber. We evaluated our hypothesis with nitrogen (N_2_) and helium (He) as two buffer gases and compared the enhancing effect of both gases in a Raman scattering intensity of H_2_. In addition, the technique was applied to propene as a VOC gas with complex Raman transitions. Propene and some of its derivatives are found in exhaled breath and could be a potential biomarker of lung cancer [36,37,38,39,40]; therefore, it was selected for the CERS experiments.

CERS technique is promising for amplifying Raman scattering intensities of challenging trace-amount molecules in breath or industrial gases. We expect CERS to become an effective Raman-based gas analysis technique with versatile applications in the analyses of breath, industrial, and environmental gas samples.

The Raman intensity enhancement offered via CERS, in addition to the enhancement provided by the FERS, can further expand the efficiency of the current gas Raman analyzers.

The aims of this study were: (1) the development of a novel Raman scattering enhancement technique, CERS, in an FERS system pumped with a nanosecond-pulsed laser; (2) the evaluation of the efficiency of Raman enhancement with two different buffer gases; and (3) the application of the technique to a VOC biomarker of lung cancer to further explore the efficiency of CERS as a Raman-enhancing technique for potential breath analysis applications.

## 2. Materials and Methods

### 2.1. FERS System Description

**Figure 1:**
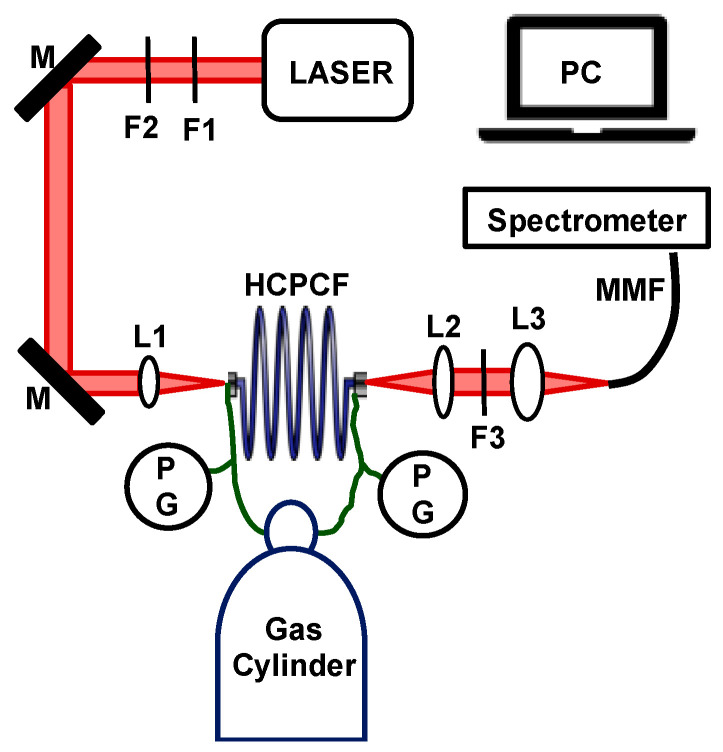
The FERS system components include F1: 785 nm laser line filter; F2: neutral density filter; M: mirror; HCPCF: hollow-core photonic-crystal fiber; PG: pressure gauge; L1–L3: lens; F3: 785 nm long pass filter; MMF: 50 µm multimode fiber.

A schematic diagram of the FERS system is shown in Figure 1. The system is a modified version of our previous system published by Chow et al. [2], where the CW pump laser was replaced with a pulsed laser for acquiring enhanced Raman spectra through SRS. Figure 1 shows the system configuration. The excitation pump beam was a dye laser pumped with a 337 nm nitrogen laser (GL-302, Horiba Scientific, Irvine, CA, USA), with a pulse duration of 0.8 ns, a pulse repetition rate of 7 Hz, and a spectral bandwidth of 0.04 nm (0.7 cm^−1^). The laser dye was Oxazine 750 Perchlorate (OD 775, Exciton, Lockbourne, OH, USA). The output of the laser was tuned to 785 nm for Raman excitation.

A 2 m long HCPCF (HC-800-B, NKT Photonics, Birkerød, Denmark) was used as the gas cell. The HCPCF had a core diameter of 7.5 ± 1 µm. The optimal transmission window of the HCPCF was from 770 to 870 nm, which allowed the excitation beam and the generated Stokes Raman photons to be transmitted in the fiber with minimal attenuation losses. Raman photons were collected in forward-scattered mode and were transferred to a spectrometer (HoloSpec™ f/2.2 Imaging Spectrograph, Kaiser Optical Systems, Ann Arbor, MI, USA) using a 50 µm multimode fiber for analysis.

All the pressures reported in this article are in absolute pressure units. The pressure of the system was monitored using two commercial generic pressure gauges (PG) with an accuracy of ± 1% in pressure reading. The system was a sealed system, and therefore, precise pressure control was important to avoid pressure drop or leakage during the measurements. A 785 nm laser line filter (F1; LL01-785-12.5, Semrock, Rochester, NY, USA) and a neutral density filter (F2) were placed in the beam path to adjust the pump pulse energy. An aspheric lens with f = 13.86 mm (L1; C560TME-B, Thorlabs, Newton, NJ, USA) was used to focus the beam into the HCPCF core. On the output of the HCPCF, the emitted photons were collimated using another f = 13.86 mm lens (L2). A long pass filter (F3; LP02-785RU, Semrock, Rochester, NY, USA) in the output beam path was used to block the laser wavelength, while transmitting the Stokes Raman lines. The transmitted emission was focused onto the multimode fiber of the spectrometer, using a third aspheric lens with f = 18.4 mm (L3; C280TMD-B, Thorlabs, Newton, NJ, USA).

### 2.2. Gas Raman Measurements

A. H_2_ Raman measurements without CERS

The FERS system was filled with H_2_ with varying pressures of 17–70 psi. Raman spectra were acquired with a pump pulse energy of 1 µJ. Raman measurements at a pressure of 20 psi were recorded as the “No CERS” base signal. These measurements served as the baseline where CERS technique was not applied. It is worth mentioning that the baseline could also be set at other gas pressures as our preliminary experiments had verified that this would not change the overall trends and conclusions.

B. H_2_ Raman measurements with the CERS technique

N_2_ as the most abundant gas in the ambient air as well as in the exhaled breath was selected as a buffer gas to examine the CERS effect. Helium, a chemically inactive gas with no characteristic Raman shift was selected as a simpler buffer gas for CERS effect.

The partial pressure of H_2_ was kept at 20 psi while N_2_ or He were added with increasing pressures, lowering the mole fraction of H_2_ to as low as 20%. Raman spectra were acquired with 1 μJ excitation pulse energy.

C. VOC Raman enhancement with CERS

Propene was chosen as the VOC to be tested with the CERS technique since it was one of the many VOCs that are found in human breath and was readily available to be purchased in gas cylinders. Raman measurements were first obtained from pure propene at a pressure of 4 psi without using the CERS technique. The pressure of 4 psi is close to the vapor pressure of some VOCs found in breath [41]. It should be mentioned that breath VOCs are mainly at much lower pressures; however, 4 psi was selected as it was much below the pressure that was tested for H_2_ Raman enhancement measurements and was easily measured within the reading range of the pressure gauges that was used. Raman spectra were then measured with CERS implemented by adding helium to the FERS system with increasing pressures of 20, 50, 80, and 110 psi. Propene partial pressure was constant at 4 psi while its mole fraction decreased to 16.7%, 7.4%, 4.8%, and 3.5%, respectively, for 20, 50, 80, and 110 psi of added helium.

### 2.3. Raman Spectral Analysis

Raman spectrum was the average of multiple Raman measurements. Raman intensities were calculated as the area of the Raman signal peaks following background subtraction. Raman intensities across gas pressure were obtained and plotted for analysis.

## 3. Results

### 3.1. H_2_ Raman Measurements without CERS

**Figure 2:**
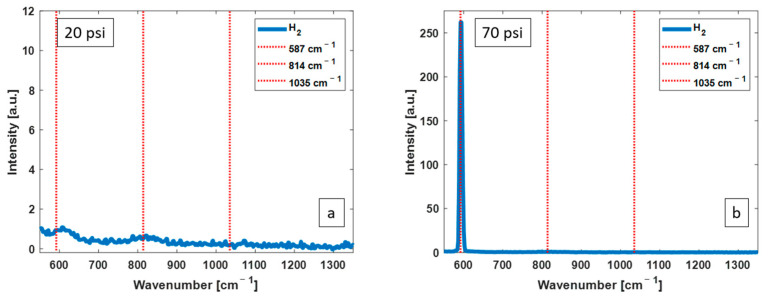
H_2_ Raman spectra without CERS at 20 psi (**a**) and 70 psi (**b**), both with an excitation pulse energy of 1 μJ. Increasing the pressure results in an increase in a Raman scattering intensity of 587 cm^−1^ Raman peak. The other Raman transitions of H_2_ are not efficiently stimulated, but their positions are shown by the red lines.

Figure 2 shows H_2_ Raman spectra without the CERS technique at two pressures of 20 psi (a) and 70 pai (b). The growth of the H_2_ Raman peak is clearly shown with increasing gas pressure. The other Raman transitions of H_2_ are not efficiently stimulated, but their positions are shown by the red lines.

**Figure 3:**
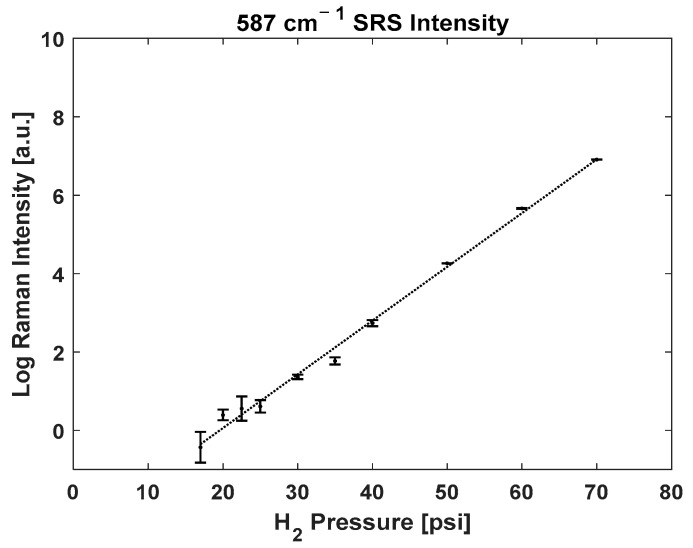
Raman scattering intensity of 587 cm^−1^ pure rotational line of hydrogen as a function of gas pressure on a natural logarithmic linear scale. Raman scattering intensity changes exponentially with increasing pressure. Note: Some error bars are too small to be shown.

Figure 3 shows how the intensity of the rotational Raman peak of H_2_ changes as a function of its own gas pressure. The data are plotted on a logarithmic linear scale. As shown by the least square regression line, an exponential function best reflects the trends of Raman intensity growth as a function of gas pressure (R^2^ = 0.99).

### 3.2. H_2_ Raman Measurements with CERS Technique

**Figure 4:**
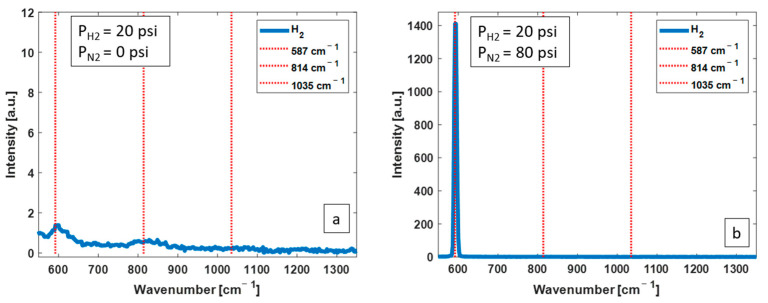
Measured Raman spectra from H_2_ at 20 psi without CERS (**a**) and with CERS (**b**) with 80 psi of N_2_ as buffer, corresponding to H_2_ mole fraction of 20%. Adding N_2_ results in a substantial enhancement of the 587 cm^−1^ Raman peak.

*N_2_ as the buffer gas:* Figure 4 shows two of the obtained H_2_ Raman spectra, without the CERS technique (a) and with the CERS technique using N_2_ as buffer (b). The partial pressure of H_2_ in both spectra is 20 psi. The mole fraction of H_2_ in (b) is 20%. There is an efficient enhancement of 587 cm^−1^ Raman peak of H_2_ when CERS is applied with N_2_ as the buffer gas.

**Figure 5:**
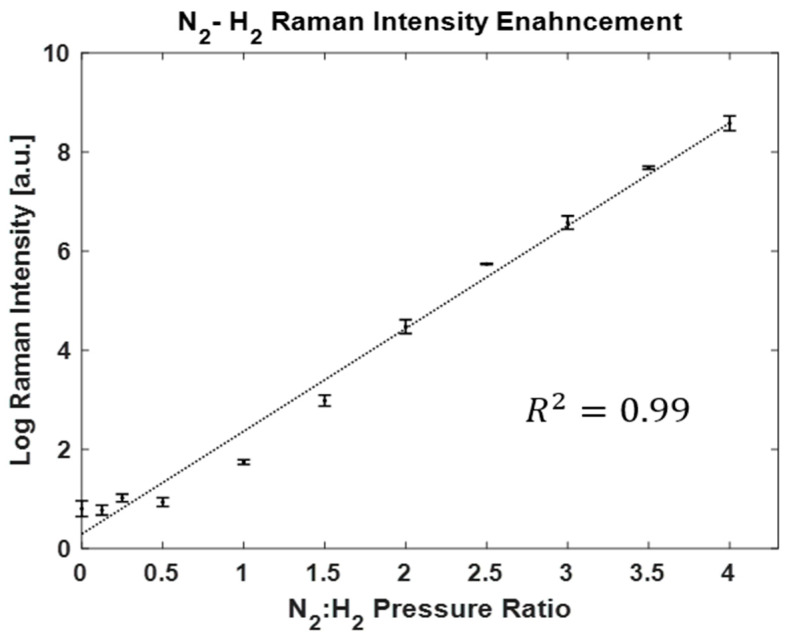
Efficient enhancement of Raman intensity with the CERS technique with increasing pressure of N_2_ gas. The *Y* axis is on a natural logarithmic scale. The trend of data shows an exponential enhancement of H_2_ Raman intensity. Note: By increasing the N_2_:H_2_ pressure ratio, the mole fraction of H_2_ decreases. The mole fraction is lowered to 20% for a N_2_:H_2_ pressure ratio of 4.

The intensity of the 587 cm^−1^ Raman line is plotted as a function of the ratio of the pressure of N_2_:H_2_ on a natural logarithmic linear scale in Figure 5. The best line of fit shows an exponential enhancement of H_2_ Raman scattering peak intensities by increasing the pressure of N_2_. With a N_2_:H_2_ pressure of 80:20, equivalent to 20% mole fraction of H_2_, there was an enhancement of the Raman intensity, up to three orders of magnitude.

**Figure 6:**
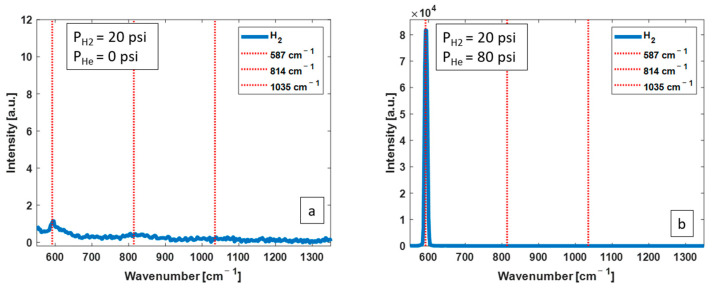
Measured Raman spectra from H_2_ at 20 psi without CERS (**a**) and with CERS with 80 psi of helium as buffer, with H_2_ mole fraction of 20% (**b**). Adding helium results in a substantial enhancement of 587 cm^−1^ Raman peak.

*Helium as the buffer gas:* Figure 6 shows two of the obtained H_2_ Raman spectra, without the CERS technique (a) and with the CERS technique using helium as buffer (b). The partial pressure of H_2_ in both spectra was 20 psi. The mole fraction of H_2_ in (b) is 20%. There is an efficient enhancement of 587 cm^−1^ Raman peak intensity when CERS is applied.

**Figure 7:**
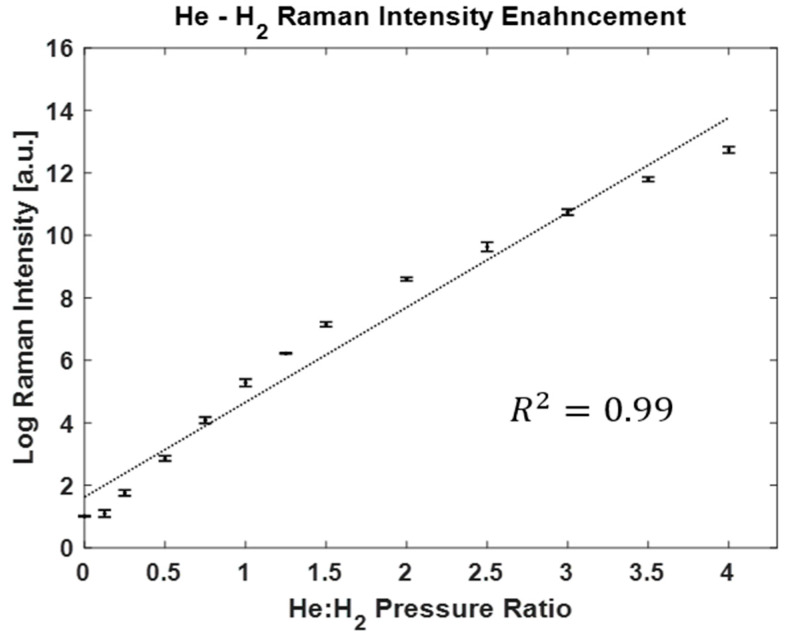
Efficient enhancement of Raman intensity with the CERS technique with increasing pressure of helium gas. The *Y* axis is on a natural logarithmic scale. The trend of data shows an exponential enhancement growth of H_2_ Raman intensity with the use of CERS technique. Note: By increasing the He:H_2_ pressure ratio, the mole fraction of H_2_ decreases. The mole fraction is lowered to 20% for a He:H_2_ pressure ratio of 4.

Figure 7 shows the magnitude of Raman scattering enhancement as a function of the ratio of He:H_2_ pressures on a natural logarithmic linear scale. There is a distinct enhancement of Raman scattering intensity by increasing the pressure of helium. With a He:H_2_ pressure of 80:20, equivalent to 20% mole fraction of H_2_, there was an enhancement of the Raman intensity, up to five orders of magnitude.

**Figure 8:**
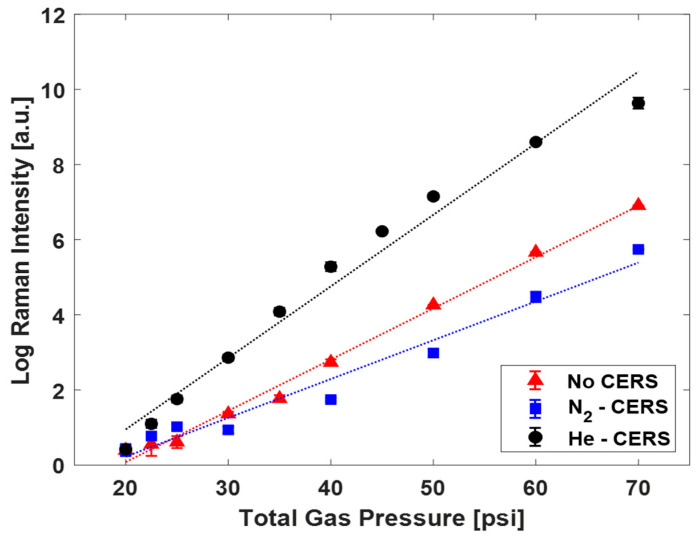
Raman intensity enhancement as a function of the total pressure. The black curve is for the CERS with He, the blue curve is for CERS with N_2_, and the red curve is for Raman measurement without CERS. Partial pressure of H_2_ is fixed at 20 psi for both the CERS with N_2_ experiment (blue curve) and the CERS with He experiment (black curve). The partial pressure for the N_2_ or He equals to the total pressure minus 20 psi (partial pressure of H_2_). As shown, the CERS with He (He-CERS) outperforms the other two enhancing mechanisms. Note: Some error bars are too small to be shown. The *Y* axis is on a natural logarithmic scale. H_2_ mole fraction is 80%, 67%, 50%, 40%, 33%, and 29%, respectively, at total gas pressures of 25 psi, 30 psi, 40 psi, 50 psi, 60 psi, and 70 psi.

Figure 8 combines data points of Figure 3, Figure 5 and Figure 7 and replotted them in a single graph for a comparison of the CERS effects generated by different buffer gases. The *X* axis has been changed to the “Total Gas Pressure”, which equals to the H_2_ pressure (fixed at 20 psi), plus the partial pressure of N_2_ or He, respectively, for the corresponding CERS with N_2_ or CERS with He experiments. The partial pressure for N_2_ or He can be calculated by taking the N_2_:H_2_ pressure ratio in Figure 5 or the He:H_2_ pressure ratio in Figure 7, multiplying by 20 psi (the fixed H_2_ pressure). For pure H_2_ experiments without CERS, the *X* axis values are the same as Figure 3. Note that only data points with a total gas pressure of ≤70 psi are included in Figure 8 in order to match the maximum H_2_ pressure shown in Figure 3.

Figure 8 facilitated evaluating the efficiency of CERS as compared to no CERS as well as comparing the enhancement effect of N_2_ versus helium. As shown, it is always more efficient to add helium to obtain enhanced Raman signals. He-CERS is even more effective than increasing the sample (H_2_) gas pressure itself.

It should be mentioned that the mole fraction of H_2_ in the “No CERS” curve (red triangles) is 100%, while the total gas pressure increases. However, the mole fraction of H_2_ (at a fixed pressure of 20 psi) in N_2_-CERS (blue squares) and He-CERS (black circles) decreases while the total gas pressure increases. The H_2_ mole fraction is 80%, 67%, 50%, 40%, 33%, and 29%, respectively, at total gas pressures of 25 psi, 30 psi, 40 psi, 50 psi, 60 psi, and 70 psi.

### 3.3. Raman Enhancement of a VOC with CERS

**Figure 9:**
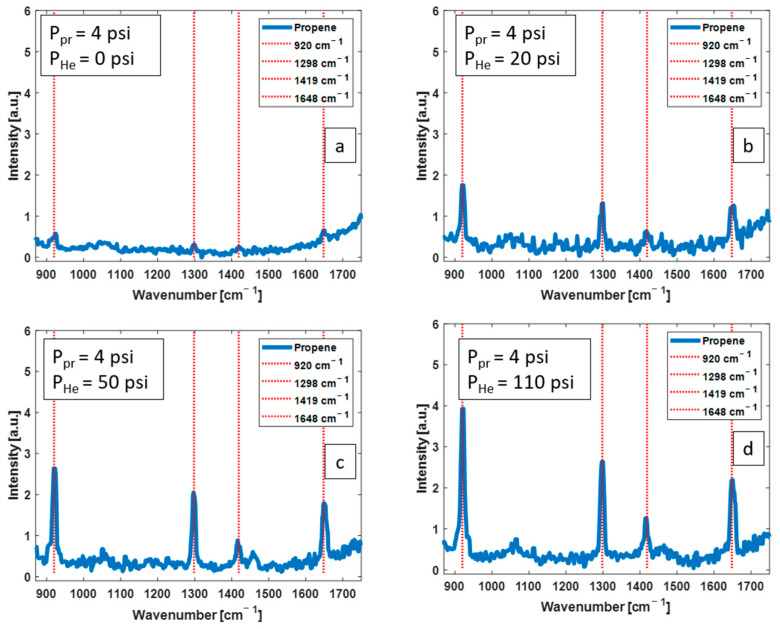
Raman spectra of propene with a partial pressure of 4 psi acquired with 1 μJ pump pulses without CERS (**a**), and with CERS using helium with pressures of 20 psi (**b**), 50 psi (**c**), and 110 psi (**d**). The partial pressure of propene is fixed at 4 psi in all Raman measurements with a mole fraction of 16.7%, 7.4%, and 4.8%, respectively, in (**b**–**d**). The intensity of Raman spectra is enhanced when He is mixed with propene due to CERS.

Raman spectra from propene were obtained without CERS (a) and with CERS (b–d) as shown in Figure 9. It is clearly seen that the intensity of Raman peaks increases when CERS is applied, and with greater enhancement with higher helium pressures. Raman spectrum without CERS has a low signal-to-noise ratio, with weak Raman peaks hidden in the noisy background signal. CERS increases the signal-to-noise ratio as qualitatively observed in Figure 9a–d.

**Figure 10:**
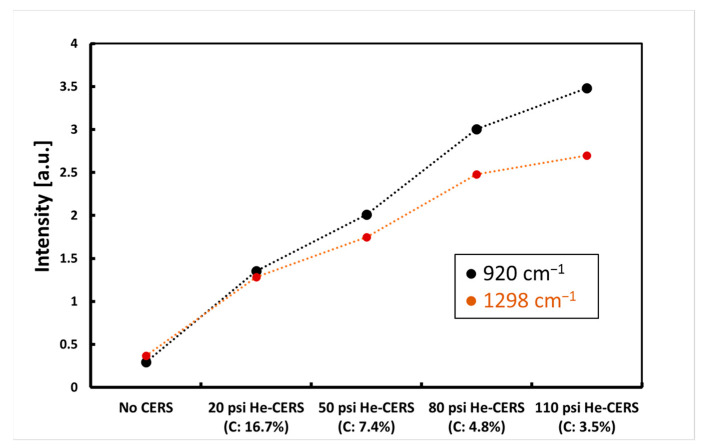
Propene Raman peak intensities without and with CERS at different helium pressures for the first two Raman peaks of 920 cm^−1^ and 1298 cm^−1^. Partial pressure of propene is 4 psi with a mole fraction of 16.7%, 7.4%, 4.8%, and 3.5%, respectively. Propene Raman scattering intensities are enhanced with increasing helium pressures in CERS.

Shown in Figure 10 are the magnitude of enhancement for 920 cm^−1^ and 1298 cm^−1^ Raman peaks of propene. Note that the partial pressure of propene is fixed at 4 psi in all the measurements. There is a considerable enhancement of Raman peak intensities when CERS is applied compared to the “No CERS” measurement. The magnitude of intensity enhancement for the 920 cm^−1^ Raman peak is approximately 5×, 7×, 10×, and 12× for CERS with 20 psi, 50 psi, 80 psi, and 110 psi of added helium, respectively. The enhancement values for the 1298 cm^−1^ Raman peak are 3×, 5×, 7×, and 7×, respectively.

## 4. Discussion

A novel Raman enhancement technique, i.e., CERS, is developed, which is based on mixing the Raman analyte gas with a buffer gas. CERS was evaluated using H_2_ as a simple gas with well-known Raman characteristics and using propene as an example VOC biomarker for lung cancer, with complex Raman transitions. Measurements were performed without CERS and with the CERS technique. As shown in Figure 4, Figure 5, Figure 6, Figure 7, Figure 8, Figure 9 and Figure 10, CERS results in a substantial enhancement of Raman peaks of H_2_ and propene. The magnitude of enhancement increases from the same amount of analyte gas by simply increasing the pressure of the buffer gas.

Two different buffer gases were examined for the implementation of CERS, N_2_, and helium with similar partial pressures. N_2_ is the most Sabundant gas in the ambient air as well as in the exhaled breath, so it was important to evaluate its effect on measured analyte gas Raman spectra. The pump energy conversion to N_2_ Raman peak of 2331 cm^−1^ is a highly inefficient process given the band gap transmission mechanism of the HCPCF. Helium is an inert gas with no characteristic Raman shifts and is therefore a Raman inactive simple buffer gas to study for CERS.

Both gases are shown to result in considerable Raman enhancement; however, helium yields more intense Raman peaks as supported by data shown in Figure 8. One incredible observation is that it is more efficient to add a buffer gas rather than increasing the number of analyte gas particles itself. This can potentially be beneficial for breath analysis applications, where the number of particles of the analyte gas cannot be increased; hence, stronger Raman spectra can be obtained from introducing a second buffer gas to the analyte gas.

Following the implementation of CERS for H_2_ Raman measurements and upon understanding that CERS with helium achieves a higher Raman signal enhancement, CERS was further tested with propene mixed with helium. As Figure 9 shows, stronger Raman spectra were obtained from propene at a fixed partial pressure of 4 psi while partial pressure of helium increases. The magnitude of enhancement was up to 12× and 7× for the 920 cm^−1^ and 1298 cm^−1^ Raman peaks of propene as compared to where no buffer gas was added (no CERS).

CERS was developed based on unexpected observations during our Raman measurements of simple simulated two-component breath samples. There could be multiple mechanisms that contribute to the Raman signal enhancement. Two main contributing mechanisms are: (1) increasing the effective interaction pathlength of the pump laser beam with the target molecules through an isotropic scattering of pump light off buffer gas molecules and (2) energy transfer between Raman active molecules and buffer gas particles through inter-particle collisions. The latter has been described by the dispersion effect in a gas laser system using buffer gas, where it increases Stokes conversion efficiency and also carefully adjusts the pressure of the buffer and target gases [42].

When a second buffer gas such as He/N_2_ is mixed with the analyte, e.g., H_2_ molecules, the pump laser beam scatters off He/N_2_ particles isotopically, which will effectively increase the excitation photon-traveling distance compared to when there is no He/N_2_ in the HCPCF. Therefore, pump photons’ trajectory will be in a zigzag pattern, with an increase in the effective pathlength. This results in more opportunity for the pump beam to interact inelastically with H_2_ molecules from all directions. The overall effect is an increase in the effective interaction pathlength of the pump photons with the analyte.

**Figure 11:**
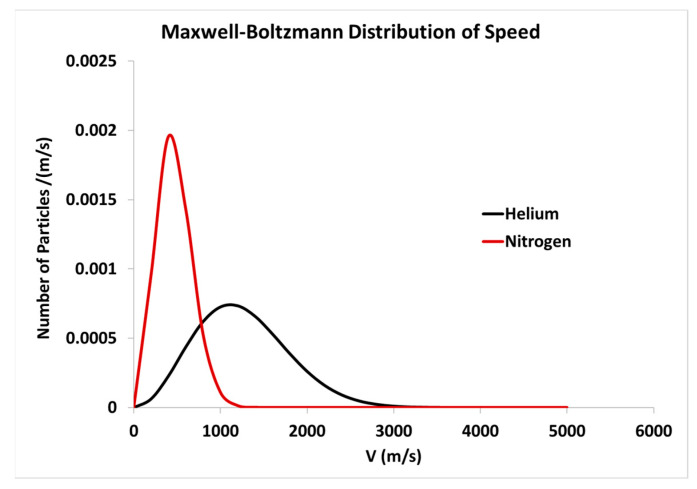
Maxwell–Boltzmann distribution of speed for He and N_2_ particles at room temperature.

The difference in enhancement between N_2_ and helium is believed to originate from the Maxwell–Boltzmann distribution of speeds of gas particles, assuming that both gases behave like ideal gases. This assumption is true under the pressures that were tested for our Raman measurements. The ideal gas assumption implies that gas particles collide elastically with other gas molecules as well as with the walls of the HCPCF, and the motion of each particle is independent of other particles. Upon colliding, particles exchange kinetic energy elastically. There are relatively more high-speed helium atoms than N_2_ molecules at room temperature as shown in Figure 11. This is derived from the root-mean-squared speed of the particles, which is an indicator of the average speed of particles of a gas ($V_{rms}=\sqrt{\frac{3k_{B}T}{m}}$), where $k_{B}$ is the Boltzmann constant, T is the absolute temperature, and m is the particle mass. $V_{rms}$ speed of He and N_2_ at room temperature are 1368 m/s and 731 m/s, respectively. This implies that the collision rate of helium atoms is higher than N_2_ molecules. Therefore, CERS is more efficient with helium than with N_2_.

Mixing an active lasing gas with a buffer gas is a common and efficient lasing enhancement mechanism, which is based on energy transfer through intermolecular collisions [43,44]. The collisional energy transfer mechanism is employed in CO_2_ lasers, for example, where helium atoms are introduced, to increase the efficiency of the CO_2_ lasing process. In atomic alkali metal vapors, the emission is amplified through mixing with helium [45,46,47] to utilize collisional energy transfer for increasing the lasing output.

The underlying enhancing mechanism in laser enhancement through mixing with a buffer gas is different from Raman enhancement through mixing with a buffer gas, as in Raman transitions energy states are virtual. However, molecular energy transfer is heuristically shown to be an effective Raman-enhancing mechanism through CERS.

Buffer gas has also been added to SRS-based gas lasers to shift the lasing wavelength [42,48,49]. These studies reported on mixing gas molecules with a buffer gas in a gas cell (laser cavity) and investigated Raman conversion efficiencies for different buffer gases under different pressures. However, none of these studies proposed using a buffer gas and target molecular collisions as a Raman-enhancing mechanism for the purpose of improving gas analysis.

Hosseini et al. [50] have shown that, when H_2_ is mixed with xenon as a buffer gas in an HCPCF, intense spectral sidebands spanning a broad range are generated. The same group has shown that [51] under a transient Raman scattering state, collisions between H_2_ and an inert buffer gas reduces the Raman gain, but results in increased Raman intensities of the first few Stokes lines. Although the underlying mechanism in their work is different from CERS, molecular collisions between the Raman gas and a buffer gas is the common enhancing mechanism. It should be noted that Hosseini et al.’s work is at a different pressure and with much higher pulse energies. They limited the buffer gas pressure to a fraction of H_2_ pressure. However, we have shown that increasing the buffer gas pressure to almost 28 times more than the Raman gas results in increased Raman intensities of the gas at a much lower partial gas pressure.

CERS can improve the Raman spectroscopy of trace-amount VOCs that are of interest in many fields, such as breath analysis for disease diagnosis and VOC monitoring in oil and gas industry. Breath tests are on the rise as they may provide a non-invasive means for the diagnosis of many diseases at early stages. However, Raman spectroscopy is not yet recognized as a sensitive analysis technique for VOC analysis as VOCs of breath are of low concentrations. CERS can improve the sensitivity of Raman spectroscopy beyond what is currently achievable with most conventional FERS techniques.

CERS has unique advantages compared to other Raman enhancement techniques in terms of simplicity and efficiency for gas analysis. Our CERS system is similar to CW-based FERS systems in terms of simplicity, with the main difference in system configuration being the use of a pulsed pump laser to replace the CW pump laser. This has improved the efficiency of Raman measurements substantially through SRS rather than spontaneous Raman scattering. On the other hand, adding a buffer gas resulted in further significant enhancement to an already enhanced SRS signal at no additional major cost to the system simplicity. Compared to CARS and conventional SRS systems with multiple-pump-Stokes input pulses, CERS is inherently a less complex system, which requires only one excitation laser source. Overall, CERS offers great advantages of simplicity and efficiency for improved gas analysis.

All Raman measurements were performed with the analyte gases trapped in a FERS system with an HCPCF as the gas cell. HCPCF offers great advantages mainly because the attenuation of light in these fibers is minimal and non-linear phenomena such as SRS occur with relatively low energy pumps. However, the micron size core of these fibers requires delicate handling and rigorous alignment. The fine-tuning of the fiber tip alignment should be routinely performed for optimizing Raman measurements and to minimize background from silica Raman interactions.

We will continue the research on CERS to extend its application for real-world gas analysis. The results presented herein are from pure gas samples. However, this improved gas analysis method can potentially be applied to more challenging gas analysis situations to improve the detection limit of FERS, which will be of interest in breath and other gas mixture component analysis applications. When more than one VOC molecules are presented in a sample, such as a breath sample from a lung cancer patient or healthy subject, the measured Raman spectra may help us to decipher the VOCs profile (relative concentration distribution of various VOCs) of the subject. A large amount of data from breath samples of thousands of subjects need to be acquired and machine learning methods (conventional statistical methods and AI methods) could then be used to analyze the acquired data and generate diagnostic algorithms. The algorithms can be used to calculate the likelihood of a subject harboring lung cancer.

## 5. Conclusions

We have successfully developed CERS, which represents a new promising Raman enhancement technique for improving gas analysis. CERS is based on mixing the Raman analysis gas with a buffer gas (nitrogen or helium) inside a hollow-core photonic-crystal fiber (HCPCF) and then measuring Raman spectra using a sub-nanosecond-pulsed laser pumping. This work demonstrated that CERS results in a substantial enhancement of the Raman scattering intensity of the analyte gas by as much as five orders of magnitude. CERS has significant advantages for improving gas analysis, with great potential for analyzing complex gases such as VOCs, which can serve as biomarkers in human breath for the screening of lung cancer and the detection of other human diseases.

## 6. Patent

The BC Cancer Institute has filed a provisional patent application related to work covered in this manuscript. The authors may obtain royalties from future commercialization of the patent.

## Data Availability

Data are contained within the article.
